# Green Synthesis of Cation Exchange Membranes: A Review

**DOI:** 10.3390/membranes14010023

**Published:** 2024-01-17

**Authors:** Stef Depuydt, Bart Van der Bruggen

**Affiliations:** Department of Chemical Engineering, KU Leuven, Celestijnenlaan 200F, B-3001 Leuven, Belgium; stef.depuydt@kuleuven.be

**Keywords:** cation exchange membranes, green membrane synthesis, material/solvent selection, PFAS, Nafion synthesis, green solvents, biopolymers, sulfonation, green chemistry, water

## Abstract

Cation exchange membranes (CEMs) play a significant role in the transition to a more sustainable/green society. They are important components for applications such as water electrolysis, artificial photosynthesis, electrodialysis and fuel cells. Their synthesis, however, is far from being sustainable, affecting safety, health and the environment. This review discusses and evaluates the possibilities of synthesizing CEMs that are more sustainable and green. First, the concepts of green and sustainable chemistry are discussed. Subsequently, this review discusses the fabrication of conventional perfluorinated CEMs and how they violate the green/sustainability principles, eventually leading to environmental and health incidents. Furthermore, the synthesis of green CEMs is presented by dividing the synthesis into three parts: sulfonation, material selection and solvent selection. Innovations in using gaseous SO3 or gas–liquid interfacial plasma technology can make the sulfonation process more sustainable. Regarding the selection of polymers, chitosan, cellulose, polylactic acid, alginate, carrageenan and cellulose are promising alternatives to fossil fuel-based polymers. Finally, water is the most sustainable solvent and many biopolymers are soluble in it. For other polymers, there are a limited number of studies using green solvents. Promising solvents are found back in other membrane, such as dimethyl sulfoxide, Cyrene™, Rhodiasolv^®^ PolarClean, TamiSolve NxG and γ-valerolactone.

## 1. Introduction

“We now live in a human-dominated world in which our increasingly extreme alterations of the environment induce increasingly extreme backlashes from nature”. This is what Morens and Fauci [1] concluded when they investigated the role of human society in the emergence of COVID-19. They found that the growth of human society in size and complexity creates an endless number of possibilities for infections to grow. The impact of the human society reaches much further than only the pathogenic world. Air pollution and climate change [2], alterations in water quality and aquatic biodiversity [3] and the release of toxic chemicals due to incidents [4] are some of the negative consequences caused by humanity. A way to break this trend was proposed by Morens and Fauci [1], i.e., that we should start to live in a more thoughtful and creative harmony with nature. Green and sustainable chemistry has the potential to create a more harmonized way of living. Many applications aimed at this green and sustainable society are in place, and many investigations are ongoing. These applications are related to the production of chemicals, the generation and storage of energy and the treatment of water, and cation exchange membranes (CEMs) play an important role in it. Examples of such applications are (i) water electrolysis, which is a process where hydrogen and oxygen gases are produced via water splitting in an electrochemical cell. Depending on the type of water electrolysis technology, the compartments are separated by CEMs or anion exchange membranes (AEMs) [5]. (ii) Artificial photosynthesis, which can produce fuels and chemicals in addition to hydrogen gas via a sunlight-induced reaction at the cathode of an electrochemical cell. This technology aims to be as circular as possible by using feedstocks such as CO_2_ and N_2_. Different types of artificial photosynthesis technologies are investigated using CEMs and AEMs [6]. (iii) Direct methanol fuel cells, in which methanol, generated via, for example, artificial photosynthesis, can be transformed into energy/electricity [7]. (iv) Electrodialysis for the desalination of brackish water or the removal of heavy metal ions from wastewater, [8,9] and (v) redox flow batteries are two more examples. The transition to a decarbonized society requires high amounts of renewable energy, demanding a high capacity for energy storage. Electrochemical energy conversion and storage systems have attracted much interest due to their high turnover efficiencies, fast response times, facile scalability and freedom from geographical constraints. Vanadium redox flow batteries operate following the principle of two vanadium-containing electrolytes undergoing redox reactions. They need a proton exchange membrane to separate the compartments, transport protons and reject the transport of vanadium ions [10].

CEMs are semi-permeable membranes for the selective transportation of cations. They contain fixed negatively charged groups that repel anions and facilitate the transport of cations when an external potential difference is applied. [11] Although these CEMs are used in applications that have the objective of making society greener, the synthesis of such membranes is not. Commercial perfluorinated membranes are synthesized by a complex, energy-intensive process using multiple solvent steps, increasing their impact on the environment [12,13,14]. Furthermore, perfluorinated membranes contribute to global pollution of per-and polyfluorinated substances (PFAS) in air, water and soil [15,16]. At this point, there are no green, sustainable alternatives to the commercial membranes because of problems related to stability, a safe and green sulfonation process [11], green and safe solvents [17] and sustainable materials [18,19].

**Figure 1:**
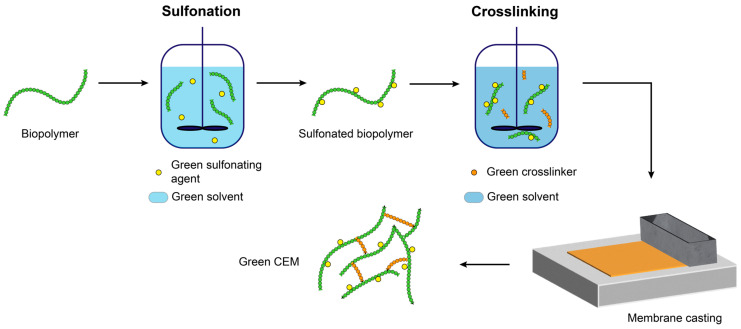
Overview of a synthesis procedure for green cation exchange membranes.

This review analyses the work that has been conducted in recent years to make CEMs greener and more sustainable. Furthermore, a discussion on the definition of green and sustainable chemistry is initiated and on the rules and tools to evaluate the ‘greenness’ and sustainability of a process. Subsequently, the synthesis of commercial perfluorinated membranes is reviewed in detail. First, the production process and the extent to which they violate the rules of green and sustainable chemistry are discussed; the related PFAS crisis will be introduced as part of this. In addition, different methods to make the production of the perfluorinated membranes greener are evaluated. Lastly, based on green sulfonation, green materials and green solvents, the production of green cation exchange membrane alternatives is discussed. These aspects constitute the main steps in the synthesis of a non-perfluorinated CEM. An example of such a procedure is given in Figure 1. Biopolymers are selected as green feedstock for the membrane polymer. The sulfonation of these biopolymers should be performed using green solvents and green sulfonating agents. To prepare the crosslinking, green crosslinkers should be selected and mixed together with the sulfonated biopolymer using a green solvent. Finally, the membrane can be cast.

## 2. Green vs. Sustainable Chemistry

In 1998, Anastas and Warner [20] were the first to give meaning to the concept of green chemistry and introduced 12 principles of green chemistry to the world. In the following years, different definitions were given: the United States Environmental Protection Agency (US-EPA) describes green chemistry as the design of chemical products and processes that reduce or eliminate the generation of hazardous substances [21]. They stress that green chemistry should be applied not only during the manufacturing phase of the product but also throughout the entire life cycle, including the design, use and disposal. Green chemistry should not be confused with remediation (clean up) that includes the treatment of waste streams. Green chemistry reduces pollution at the source [21]. Lewandowski [22] explains that the goal of green chemistry is to design (or redesign) products and manufacturing processes to reduce their impact on human health and the environment. He states that fundamental to green chemistry is the idea of sustainability, reducing environmental impacts and conserving natural resources for future generations. The concept of sustainable chemistry came to life more recently in the second decennium of the 21st century and received multiple different definitions from companies, governmental entities, etc. [23]. Kümmerer [24] said that chemistry can only be called sustainable when it does not have any drawbacks for the environment, safety and health. Using the definitions for green and sustainable chemistry mentioned above, it is clear that sustainable chemistry is more than green chemistry. Sustainable chemistry includes green chemistry [25]. The professional evaluation and certification board, also referred to as PECB, [26] explains the difference by stating that a green method uses environmentally friendly products and services, whereas sustainable methods use them without damaging the resources of future generations. Green chemistry makes processes and products greener, but it lacks quantitative standards, whereas sustainable chemistry is clearer and sets a hard standard that the chemistry should not have any negative consequences for the environment, health and safety.

In 2021, the United Nations shared a manual framework for green and sustainable chemistry [27]. In this framework, they promote the development of greener and more sustainable chemistry innovations. They state that it can play a significant role in achieving Sustainable Development Goals (SDGs). A large number of SDGs would directly benefit from it, such as zero hunger (SDG 2) and sustainable consumption and production (SDG 12). They also included ten objectives and guiding considerations for what green and sustainable chemistry seeks to achieve, which are listed in Table 1.

There are similarities between the 10 points of the UN and the 12 principles of green chemistry of Anastas and Warner [20], i.e., both frameworks give importance to the usage of safe, non-toxic chemicals and they both aim for the minimization of waste and pollution prevention. The framework of the UN takes safety one step further by also giving particular attention to the possible negative trade-offs of substitutions or alternatives for toxic products. Furthermore, the UN extends its framework with a wider social aspect by including high standards of ethics, education and justice in chemistry innovation. In the remainder of their report, they explain how stakeholders can evaluate the green and sustainable performance of their chemicals/chemistry. To evaluate the hazards of chemicals, a globally harmonized system for the classification and labeling of chemicals is a tool that helps in the construction of a chemical hazard assessment. Gauthier et al. [28] examined 32 chemical characterization tools and compared them based on five different criteria. They did not find one tool scoring high in all five categories and only a few tools characterized risks. This shows the importance of having proper knowledge of how to work with these tools while constructing a hazard assessment. Furthermore, the UN discusses three quantitative metrics that can help in evaluating the green and sustainable chemistry character: (i) The E factor equals the generated mass of waste divided by the generated mass of product. The lower the E factor is, the more environmentally benign it is. (ii) The process mass intensity index goes one step further and takes into account all the materials used to produce the chemicals. The process mass intensity index is defined as the total mass of the material needed and used to produce a specified mass of the product. Jimenez-Gonzalez et al. [29] discussed the importance of this index in the pharmaceutical industry; they showed that the cumulative environmental impact of producing the pharmaceuticals is much higher than the impact of the waste produced. (iii) When there is a need to quantify the amount of used chemicals of high concern, the Chemical Footprint method can be used to measure the progress in the reduction of these chemicals.

The reason why it is important to follow the principles and objectives of green and sustainable chemistry is to eliminate risks and hazards of all kinds as much as possible. This method can be applied to the synthesis ofCEMs. At this point, the principles of sustainable chemistry are not followed, resulting in negative consequences: (i) The traditional sulfonation processes use (fuming) sulfuric acid, which generates a substantial volume of acid waste [11], violating the principle of waste prevention and the objective of ‘advancing the sustainability of production processes’. This violation leads to large waste streams that need to be treated, thereby demanding more energy and contributing to climate change. (ii) The principles and objectives related to hazardous chemicals are violated since perfluorinated polymers are the main polymers used in commercial CEMs, and toxic solvents are used to dissolve them. The use of these polymers contributes to the PFAS crisis and has already caused multiple incidents with fatal casualties [30,31]. The PFAS crisis directly shows the importance of following the principles of ‘design for degradation’ and ‘real time pollution prevention’. More discussion on the hazards is provided later. (iii) The conventionally used materials to synthesize CEMs, such as polyvinylidene fluoride (PVDF), polyether ether ketone (PEEK), polyether sulfone (PES) and polysulfone (PS), make use of non-sustainable fossil feedstocks. The use of these kinds of feedstock is in direct violation of the ‘use of sustainable feedstock’ principle for green chemistry. However, the problems associated with using these feedstocks go beyond only one principle, i.e., fossil fuel-based polymers, which also contribute to three major threats of our time: climate change, plastic pollution and loss of biodiversity [32].

## 3. Greenness and Sustainability of Perfluorinated Membranes

**Figure 2:**
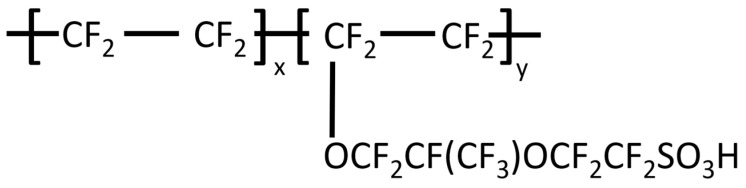
Chemical structure of Nafion. Nafion is fully perfluorinated and consists of a hydrophobic backbone and a hydrophilic side chain with a sulfonic acid group.

Perfluorinated cation exchange membranes such as Nafion are the most widely used CEMs nowadays [6,33,34]. Nafion was developed by Dupont in the late 1960s to serve in chloralkali cells for the production of NaOH, KOH and Cl_2_. In the process, Nafion has to prevent the back migration of OH^−^ ions, keep Cl_2_ and H_2_ separate and facilitate the transport of Na^+^ ions [35,36,37,38]. In such a process, chemical and thermal stability and high cationic conductivity are crucial. Nafion fulfills these requirements, and Figure 2 shows its polymeric structure [39]. The combination of the hydrophobic backbone with pendant sulfonic acid groups on the hydrophilic sidechains creates a nanophase separation that leads to high conductivity. In addition, Nafion’s perfluorinated structure gives high thermal and chemical stability. The combination of its strong acidity, chemical and thermal stability and high conductivity is the reason why Nafion expanded its application range to many other applications such as acid catalysts, membranes in electrosynthesis and as a separator in fuel cells [35,36,40]. The synthesis of Nafion membranes was patented in the 1960s by Dupont [38,41,42]; now, these patents have expired, and other companies brought their own perfluorinated CEMs with sulfonic acid groups to the market. Examples of such popular competitors of Nafion are Flemion (AGC Chemical, Exton, PA, USA), DowMembrane (Dow Chemical, Midland, MI, USA) and Aciplex (Asahi Chemical, Tokyo, Japan) [14].

When taking a closer look at the synthesis process of these perfluorinated membranes, the story is not that bright. These polymers are manufactured via a complex and solvent-intensive process, making them neither green nor sustainable [12,38,41,42,43]. To clearly show the complexity of the synthesis process, a summary of the synthesis method is given based on the patent claims by Dupont in the 1960s [38,41,42,43]. This synthesis procedure is used to verify the extent to which Nafion follows the principles of green chemistry of Anastas and Warner and the 10 sustainable objectives from the United Nations. The Nafion polymer is formed via the copolymerization of tetrafluoroethylene and a perfluoro (alkyl vinyl ether) with sulfonyl acid fluoride. The copolymerization step is preferably performed using sulfonyl acid fluoride (−SO_2_F) rather than sulfonic acid (−SO_3_H) to avoid side reactions [14]. The sulfonyl acid fluoride group is converted into a sulfonic acid group via reaction with KOH in dimethyl sulfoxide (DMSO) [36]. The two main industrial methods to produce tetrafluorothylene are based on the pyrolysis of trifluoromethane and chlorodifluoromethane. Tetrafluoroethylene should be treated with care; it can explode with oxygen at low temperatures, and even in the absence of oxygen, it can decompose and explode [44]. The synthesis of perfluoro (alkyl vinyl ether) is more complex. The monomer is obtained via the pyrolysis reaction of its corresponding acid fluoride or via the pyrolysis of the alkali metal salt of its corresponding carboxylic acid. The pyrolysis process occurs at 200–600 °C. For the pyrolysis of the corresponding acid fluoride, a metal oxide such as zinc oxide or silica is used as a solid catalyst for the gas phase reaction. The alkali metal salt of the corresponding carboxylic acid is formed via the reaction of the corresponding acid fluoride with an alkali metal salt of a weak acid. The synthesis of the corresponding acid fluoride is obtained via the reaction between hexafluoropropylene epoxide or tetrafluoroethylene epoxide and alpha-fluorosulfonyl perfluoroacyl fluoride. Solvents with the structure XC_p_F_2p_CH_2_Cl (X = halogen or hydrogen; 1 < *p* < 10) or methylene chloride, chloroform or 1,2-dichloroethane can be used. Tetrafluoroethylene epoxide is prepared via the oxidation of tetrafluoroethylene using an aqueous alkaline hydrogen peroxide. The synthesis of alpha-fluorosulfonyl perfluoroacyl fluoride is described in the patent of England et al. [43].

Although the summary of the synthesis did not give all the details on the synthesis procedure, the process did not follow the principles of Anastas and Warner, eventually leading to it not being green or sustainable, resulting in negative consequences: (i) Hazardous chemicals are used in multiple steps of the synthesis process: tetrafluoroethylene, which is a very explosive character and possibly carcinogenic to humans (Group 2B), and KOH, which is corrosive and causes skin burns. Working with these chemicals can lead to severe accidents and it already did. In 1999, a plant producing polytetrafluoroethylene from tetrafluoroethylene exploded, resulting in three deaths [31]. More recently, in 2021, the employers of a plant dealing with fluorocarbon chemicals were exposed to these toxic gases. Two employers died from respiratory problems, and a third employer needed intense treatment in the hospital [45]. These accidents raise the question of whether it is even possible to work risk-free when using such dangerous chemicals, despite the existence and implementation of all kinds of safety measurements. Only eliminating the use of such hazardous chemicals would eliminate all the risks. (ii) Toxic solvents such as chloroform or 1,2-dichloroethane are also used. Since these solvents are carcinogenic, severe health risks are also present when using these chemicals. (iii) energy efficiency: high temperatures are necessary for the different pyrolysis steps, leading to high energy demand and greenhouse gas emissions. (iv) No renewable feedstocks, but petrochemical sources are used, depleting fossil fuel reserves. (v) The high thermal and chemical stability makes it very difficult for Nafion to degrade, contributing to the PFAS crisis. The PFAS crisis and the role of perfluorinated membranes are discussed in detail later. Since Nafion is not able to meet the 12 principles of green chemistry, it worsens the 10 sustainability objectives of the United Nations. In addition, the story is not brighter for the commercial alternatives to Nafion, which are also perfluorinated membranes. To synthesize Flemion for example, hazardous chemicals are used, such as oleum; furthermore, iodine-containing waste is generated [13].

Another problem not yet discussed concerning the ‘greenness’, sustainability and even safety of the manufacturing and use of perfluorinated membranes is the generation and release of PFAS into the environment. PFAS stands for per- and polyfluoroalkyl substances. These are man-made chemicals containing carbon-fluorine bonds. Because of the strong characteristics of these bonds, they resist degradation in the environment. The pollution of PFAS has already spread all over the world in the air, groundwater, surface water and soil. They are also found in humans and have accumulating characteristics. Certain PFAS are toxic for reproduction and can harm the development of fetuses, and several PFAS may cause cancer in humans [15,16,46]. It can be concluded that perfluorinated membranes contribute to pollution in every stage of their life. Feng et al. [47] mimicked high-temperature operating conditions in which Nafion is applied and they observed the thermal degradation mechanisms. They found that these operating conditions caused the cleavage of the backbone and side chains, degrading the membrane. This degradation can occur during the operation and during the incineration phase at the end of life, indicating that Nafion is a high potential candidate for PFAS pollution. Chen et al. [48] and Zhou et al. [30] measured the concentration of PFAS in air, outdoor dust, groundwater and surface water around plants manufacturing fluoropolymers. Both studies were conducted at completely different geographical locations (USA vs. China) and both studies concluded that higher PFAS concentrations were found in areas closer to the plant, making the production plants a source of PFAS pollution. The observations of previous studies show that the manufacturing and the usage of perfluorinated membranes have the potential to cause and indeed cause PFAS pollution. Therefore, a restriction proposal on PFAS-containing chemicals was submitted by five European countries (The Netherlands, Denmark, Germany, Norway and Sweden) on 13 January 2023 [46]. Hydrogen Europe showed their concerns about this restriction in their position paper in January 2023 [49]. Hydrogen Europe is the European association representing the interest of the hydrogen industry and its stakeholders and promoting hydrogen as an enabler of a zero-emission society. They underline the fact that at this point the fluoropolymers are essential in proton exchange membrane fuel cells because there is no alternative that comes close to the same key performance indicators. A complete ban on these chemicals would threaten the competitive characteristics of the entire European hydrogen industry. Instead of a complete ban, they plead to make it possible for users to keep using the fluoropolymers if they do not have an alternative available with the same key performance indicators. For exempted users, a framework should be set up explaining the best practices of how to handle chemicals in the different stages of their life. Secondly, they plead for extra research into finding non-fluoropolymer alternatives. Although the suggestions of Hydrogen Europe can improve the situation, especially concerning material handling during manufacturing, they do not ensure a transition to green and sustainable perfluorinated membranes. Therefore, significant further improvements are required. Over the years, improvements have been made to make the synthesis process greener. Okazoe describes in his review paper different improvements in fluorination on a manufacturing scale [50]. A key improvement is the development of direct fluorination using liquid F_2_. Okazoe et al. [13] found a way to synthesize fluorinated chemicals starting from their corresponding non-fluorinated alcohol. His procedure makes it possible to change the synthesis process of Flemion, eliminating the use of toxic oleum and eliminating an additional solvent. Despite improvements in the synthesis of perfluorinated polymers, the procedure is not green and far from sustainable. The intrinsic non-degradable character of these polymers makes it very difficult, even in theory, to make them sustainable. Therefore, it is crucial to explore completely different materials, such as aromatic polymers and polyvinyl alcohols, which do not have this perfluorinated structure and have different synthesis procedures. It is important to note that although there is no alternative with the same key performance indicators as the perfluorinated membranes on a commercial scale, multiple studies reported in the literature have shown that it is possible to achieve even higher performances on a lab scale. For example, the use of polybenzimidazole-based CEMS for high-temperature proton exchange membrane fuel cells yields the highest current density and peak power density due to their high catalyst activity and molecular dynamics, proving that the perfluorinated structure can be replaced [51,52]. The synthesis of the majority of these alternatives consists of three major blocks: sulfonation, material selection for the backbone and crosslinker and solvent selection. These three aspects are discussed in the next sections.

## 4. Green Sulfonation

Similar to the commercial perfluorinated membranes, the traditional functional groups in CEM polymers that are responsible for the cationic conductivity are sulfonic acid groups (SO_3_H-groups). In the case of aromatic sulfonation with, for example, sulfuric acid, the reaction is an electrophilic substitution reaction. First, sulfuric acid dissociates into SO_3_ and H_2_O, and in the second step, an electron-rich carbon from a benzene ring binds with the sulfur of the SO_3_, forming SO_3_H groups [53,54]. The traditional way of introducing these groups uses strong oxidative agents such as fuming sulfuric acid, sulfuric acid, chlorosulfonic acid and fluorosulfonic acid. These chemicals directly violate the principles of green chemistry and the objectives of green and sustainable chemistry related to the hazards of chemicals and the protection of employees. In addition, a large amount of acid waste is generated, which is hazardous to the environment [11,55,56]. Another common way to perform aromatic sulfonation involves the used of sulfur trioxide directly. This method has multiple advantages over the other sulfonation methods: (i) fast reaction, (ii) no waste acid generation, (iii) no corrosion to equipment, and (iv) not too many byproducts formed since SO_3_ attaches as a whole to a substrate; it does not originate from the dissociation of a product. Thus, the generation of water is avoided. These advantages make it greener than the previously mentioned alternatives. However, it should be mentioned that sulfonating with SO_3_ produces heat. Additionally, this method is prone to incidents since it is not always easy to control; for example, when liquid SO_3_ is used, the viscosity increases significantly over time [11,57]. However, attempts using SO_3_ are reported in the literature with the aim of making the sulfonation process greener. Xu et al. [58] synthesized hexadecylbenzene sulfonic acid in a continuous stirred-tank microreactor using SO_3_ dissolved in 1,2-dichloroethane. The dilution of SO_3_ in a solvent makes the process easier to control due to better heat dissipation. The use of a microreactor makes the process greener because fewer material is needed, and the reaction can be performed with very low residence times (10 s). An important disadvantage of this process is the use of a toxic organic solvent; however, there are possibilities of replacing 1,2-dichloroethane with a safe, green solvent, which will be discussed in Section 6. Another way to make the process more controllable is by using SO_3_ in the gas phase [56]. Kucera et al. [59] sulfonated solid polystyrene beads with gaseous SO_3_. This method does not have any sustainability issues with solvents because there are no such issues. Furthermore, they were able to obtain high ion exchange capacities to the point where they could dissolve the sulfonated polystyrene in water. The non-optimality of this process is related to the diffusion limitations of SO_3_ gas in the beads. They observed that a sulfonated polystyrene layer was formed on the outside of the bead. It is important, however, that the sulfonic acid groups are present throughout the entire cross-section of the final membrane such that conducting channels can be formed.

A second way of introducing sulfonic acid groups to the membrane polymer is to use monomers that already include those groups. This approach is really common when polyvinyl alcohol (PVA) is used as a backbone polymer since it does not have aromatic rings on which SO_3_ can react via electrophilic substitution. A significant advantage of using PVA as a membrane polymer is that water can be used as the solvent. Wang et al. [60] sulfonated PVA by the addition of sodium vinyl sulfonate (25 wt%). Therefore, the PVA was first deprotonated using NaOH. This method makes use of very low-hazard chemicals because water is used as the solvent and the 25 wt% sodium vinyl sulfonate solution is not identified as a hazardous substance [61]. However, when investigating how green a process is, the origin of the chemicals also needs to be taken into account. Pure vinylsulfonic acid is a chemical corrosive to metals and skin [62]. The production of that chemical occurs in two steps. In the first step, carbyl sulfate is produced via an exothermic reaction between ethylene and sulfur trioxide. In the second step, this carbyl sulfate is transformed into vinyl sulfonic acid by alkaline hydrolysis [63,64]. This shows that even when sulfonated monomers are used, the traditional sulfonating agents that were discussed before are not eliminated, but they just shift to another step in the complete process. The advantage of the production of vinylsulfonic acid is that the sulfonation reaction occurs in the gas phase, which gives easier control than a reaction in the liquid phase. One disadvantage is the explosive behavior of carbyl sulfate, thereby ruining the greenness of the process. To make this process more green/sustainable, process intensification should try to alter the chemistry to avoid intermediate product formation. Thakur et al. [65] avoided the use of strong oxidants by choosing already sulfonated monomers to synthesize CEMs. They used 2-acrylamido-2-methylpropane sulfonic acid and attached it to polyacrylic acid backbones via free polymerization. However, to synthesize 2-acrylamido-2-methylpropane sulfonic acid, there is a need for sulfuric acid [66]. Another sulfonated monomer, whose synthesis can be traced back to sulfuric acid, is lithium 4-vinylbenzenesulfonate, since it is a sulfonated aromatic compound. This compound has proven its potential as a CEM for electrodialysis [67]. A popular way of performing sulfonation and crosslinking of PVA backbones together is with the use of sulfosuccinic acid. Ngo et al. [68] used sulfosuccinic acid together with glutaric anhydride to crosslink PVA backbones. They used water as a solvent and synthesized green cation exchange membranes with high ion exchange capacities. Due to the crosslinking, the water swelling even decreased with increasing ion exchange capacity. However, the synthesis becomes less sustainable when the production process of sulfosuccinic acid is taken into account. Sulfosuccinic acid is corrosive [69] and it is produced by the reaction of maleic acid with fatty alcohols and sodium bisulfite as a sulfur source [70]. The problem again arises when looking at the sulfur source in more detail, and sodium bisulfite is industrially produced from sodium sulfite and SO_2_ gas [71,72]. SO_2_ gas is listed as an acutely toxic, corrosive and irritant, which violates the principles of green chemistry. Deboli et al. and Firganek et al. both used 3-sulfopropyl methacrylate as an already sulfonated monomer to make CEMs with a densely crosslinked network. They both put effort in making their synthesis procedure greener by eliminating the solvent or by using a greener solvent, however, the production of sulfopropyl methacrylate is not environmently friendly. It is synthesized by a reaction between the corresponding methacrylic salt and 1,3-propane sultone or 1,4 butane sultone, both of which can cause cancer [73,74,75].

**Figure 3:**
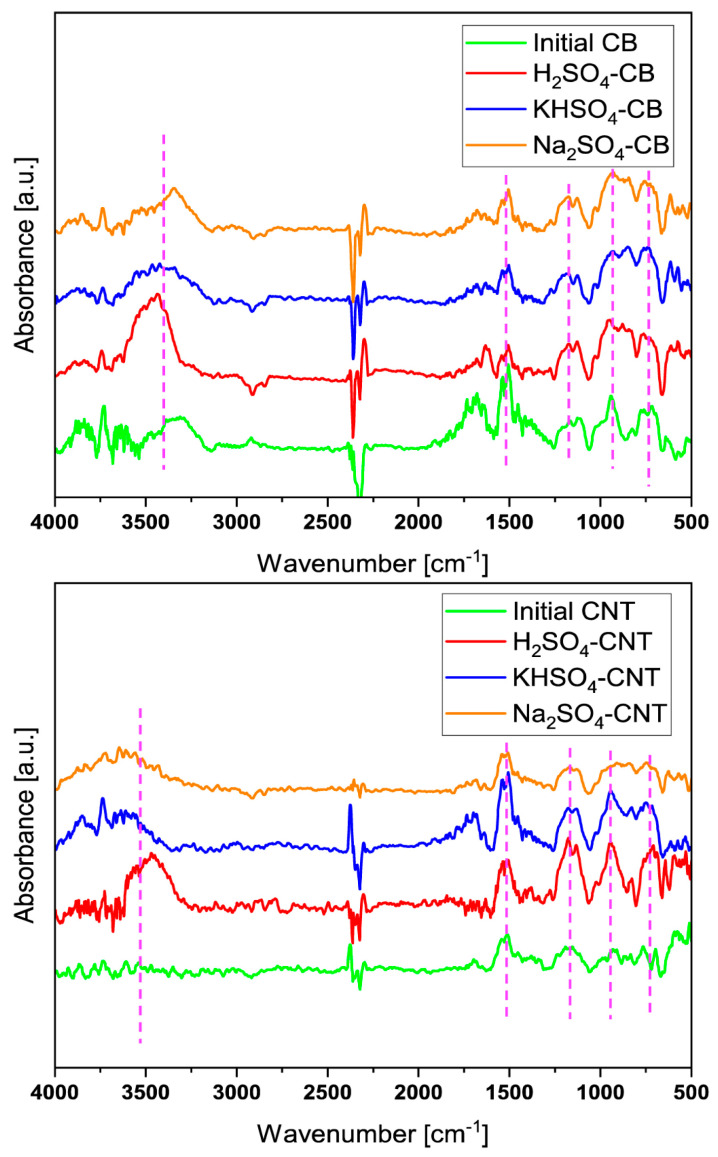
FTIR spectra of carbon black (CB) and carbon nanotubes (CNT) sulfonated using gas–liquid interfacial plasma under atmospheric pressure conditions. From Deng et al. [77] ©IOP Publishing. Reproduced with permission. All rights reserved.

A third way of introducing functionality in the form of sulfonic acid groups in the membrane polymer is to use a filler material. Filler materials in cation exchange membranes are often used to enhance properties such as mechanical and thermal stability, methanol rejection and conductivity. Furthermore, the manner in which the filler material is distributed is an extra degree of freedom that can be used to tailor the performance of the CEM. The majority of the literature reports on homogeneously spread filler materials. However, non-homogenous distributions have also been discussed in recent papers. Sun et al. [76] prepared inorganic–organic CEMs consisting of tungsten oxide nanofiller in a Nafion matrix. They observed a non-homogeneous distribution of the filler material: a “Nafion-rich layer” and “Tungsten oxide-rich layer” were present, separated by a clear interface. The introduction of this filler material following that distribution resulted in better performances such as a higher Coulombic efficiency, higher energy efficiency and higher capacity retention. Other examples of filler materials are carbon nanotubes, silica particles, titania nanotubes and nanowires [77,78]. These types of 1 D filler material have the capability of easily forming a 3 D network in the bulk polymer, forming an additional efficient conductive network when sulfonated. This network is more efficient than the traditional networks formed by the sulfonic acid groups covalently bound to the polymer network because of the lower tortuosity of the conducting channels formed by the filler material [77]. Multiple different types of filler materials have been used to enhance the cation exchange performance such as sulfonated graphene oxide nanotubes [79,80,81,82,83], sulfonated silica particles [84,85], titania nanotubes [86] and carbon nanotubes [87]. Fan et al. [88], for example, developed CEMs for electrodialysis using sulfonated poly(2,6-dimethyl-1,4-phenyleneoxide) as a polymer matrix and sulfonated carbon nanotubes as filler materials. The addition of 20 *w*/*w*% to these sulfonated carbon nanotubes lowers the ion exchange capacity (IEC) of the membrane from 1.84 meq/g to 1.48 meq/g due to its lower sulfonation degree than that of the backbone. However, the area resistance is also reduced from 1.39 Ω cm^2^ to 1.19 Ω cm^2^, showing a more efficient conducting channel of the sulfonated carbon nanotubes. Furthermore, the addition of carbon nanotubes does not affect negatively the permselectivity, which remains constant (86.2% vs. 84.5%). However, conventional methods by which these types of filler materials are sulfonated are not green. They rely on the use of strong oxidants such as fuming sulfuric acid, concentrated sulfuric acid and propane sultone. A way to improve the ‘greenness’ of sulfonation is found in the catalysis. Qin et al. [89] synthesized sulfonated carbon nanotubes, reduced graphene oxide and activated carbon using 1 M H_2_SO_4_ as a sulfonating agent, which is a step forward because strong oxidants are also used in the catalysis field as conventional sulfonating agents [77,89]. Qin et al. [89] performed sulfonation via gas–liquid interfacial plasma reaching sulfonic acid and total acid group densities of carbon acid catalysts in the range of 0.36–0.59 mmol/L and 3.47–3.63 mmol/g, respectively. Deng et al. [77] investigated this reaction mechanism. They showed that a high gas temperature (>1050 K) in the plasma zone together with the electron impact causes the generation of several active species originating from H_2_SO_4_, such as •OH, SO_3_ and HOSO_2_•. These active species graft on the defects of the carbon material, giving it a sulfur content. They also investigated the possibility of using KHSO_4_ and Na_2_SO_4_ as sulfonating agents in addition to H_2_SO_4_. Unfortunately, at this point H_2_SO_4_ still leads to sulfonation contents that are clearly higher (H_2_SO_4_: 0.36 mmol/L, KHSO_4_: 0.12 mmol/L Na_2_SO_4_: 0.06 mmol/L). The FTIR spectra of carbon black (CB) and carbon nanotubes (CNT) treated via gas–liquid interfacial plasma under atmospheric pressure conditions obtained by Deng et al. are shown in Figure 3. The peaks at 1180 cm^−1^ and 895 cm^−1^ correspond, respectively, to the stretching vibrations of the O=S=O bonds and of the C-S bond. Besides the more enhanced effect of using H_2_SO_4_ for sulfonation, carbon nanotubes have been shown to be the better filler material for this method. In addition, it is important to note that these sulfonation methods target defects found in the material’s lattice, indicating that the method is suitable for materials investigated by Qin et al., but not for polymers. To evaluate the ‘greenness’ of the sulfonation procedure, it is not only important to take a look at the sulfonation step but also at how the filler materials are fabricated. Titania nanotubes rely on the use of high concentrations of NaOH [86] and for the fabrication of graphene oxide and reduced graphene oxides, strong oxidants are required [90,91]. Therefore, the use of sulfonated carbon nanotubes has the highest potential of being green at this point. Another interesting sulfonating agent besides H_2_SO_4_ is 3-mercaptopropyltri-methoxysilane (MAMS) due to its low risks while handling the material. The agent contains S-H groups that are transformed into sulfonic acid groups after oxidation in hydrogen peroxide [83,86,92,93]. Jun et al. [86] sulfonated titania nanotubes using MAMS; something that needs to be taken into account is that the green synthesis of these titania nanotubes is not on point yet. At this point, a solution of 10 M NaOH is necessary for the synthesis. Zhongqing et al. [83] and Chowdurry et al. [92] grafted MAMS onto graphene oxide particles. Chowdurry et al. enhanced the proton conductivity of their graphene oxide membranes using MAMS. They obtained very high in-plane conductivities of 7.05 mS/cm. However, the graphene oxide membranes showed anisotropy due to the spreading of the functional groups, which limited the through-plane conductivity to only 0.19 mS/cm, whereas Nafion had a through-plane conductivity of 1.23 mS/cm. Future research should focus on removing this anisotropy. Furthermore, Mosa et al. [93] succeeded in introducing MAMS into a membrane without grafting it onto a filler material. They attached MAMS via polymerization with 3-glycidoxypropyl trimethoxysilane to polyvinyl alcohol (PVA) backbones, resulting in membranes with high conductivity.

An important aspect that has to be taken into account is the risk of leaching of the filler material (for example, nanoparticles) and polluting other systems. Kajau et al. [94] investigated the effect of prolonged exposure of polyethersulfone membranes incorporated with CuO nanoparticles to different cleaning solutions. They noticed that the leaching of nanoparticles took place after 840 h of exposure, which also changed the characteristics of the membrane. Leaching of the filler material directly violates the principles of green and sustainable chemistry related to pollution and risks and must be avoided. One way to avoid this is by attaching it to larger agents within the membrane matrix that do not leach that easily. Mahmoud et al. [95] attached Ag nanoparticles to graphene oxide nanoplates embedded in the membrane matrix. They noticed that this technique also created an even distribution of nanoparticles and that no agglomerates were formed. Hanif et al. [96] immobilized Ag nanoparticles using relatively green materials, i.e., by using tannic acid to immobilize them on a cellulose membrane. They observed no leaching during the anti-leaching test.

**Figure 4:**
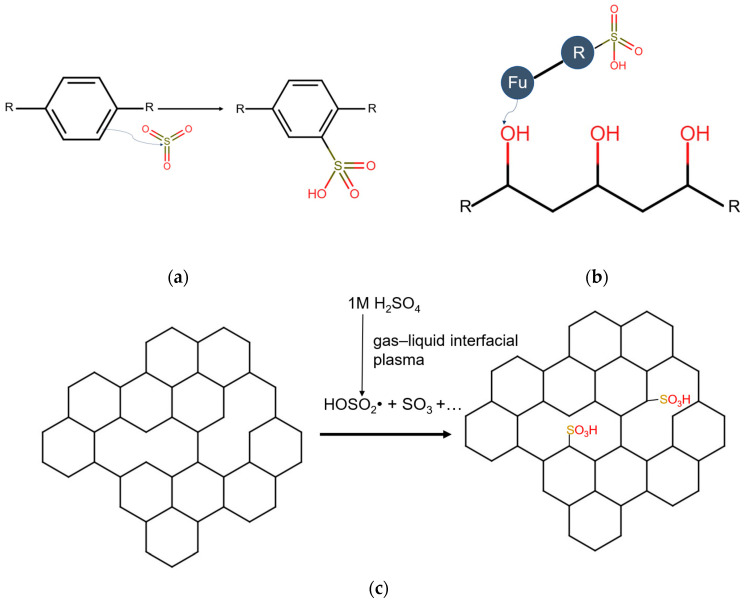
Three concepts of how materials can be sulfonated: (**a**) Direct sulfonation via electrophilic substitution of an aromatic polymer backbone using SO_3_. The SO_3_ originates from gaseous SO_3_, concentrated sulfuric acid or fuming sulfuric acid. (**b**) Sulfonation using an already sulfonated monomer. In this example, the functional group of the monomer reacts with the hydroxyl group of a polyvinyl alcohol backbone. The functional group of the monomer decides which kind of bond is formed. (**c**) Reactive species are formed using the gas–liquid interfacial plasma technology. These species react with defects in the filler material.

The three discussed concepts of how materials can be sulfonated are shown in Figure 4.

## 5. Green Materials

The conventionally used materials to synthesize CEMs, such as polyvinylidene fluoride (PVDF), polyether ether ketone (PEEK), polyether sulfone (PES) and polysulfone (PS), make use of non-sustainable fossil feedstocks. The use of these kinds of feedstocks is a direct violation of the ‘use of sustainable feedstock’ principles for green chemistry. However, the problems associated with the use of these feedstocks go beyond only that one principle, i.e., fossil fuel-based polymers also contribute to three major threats of our time: climate change, plastic pollution and loss of biodiversity [32]. In addition, it is assumed that real long-term solutions always have to be in a circular and steady state. Removing millions of tons of crude oil from the ocean floor without refilling is not a long-term solution. Therefore, the interest in natural biopolymers has increased significantly in recent years. These polymers are derived from natural raw materials, such as plants, animals or microbial biomass, making them a sustainable and renewable feedstock. In addition, most of these polymers have no or very low toxicity and are biocompatible and biodegradable [18,19,97,98]. These characteristics are the complete opposite of the commercial perfluorinated polymers, which eliminate incidents such as the discussed PFAS pollution. This review discusses five biopolymers showing the potential to synthesize CEMs: chitosan, cellulose, polylactic acid, alginate and carrageenan.

**Figure 5:**
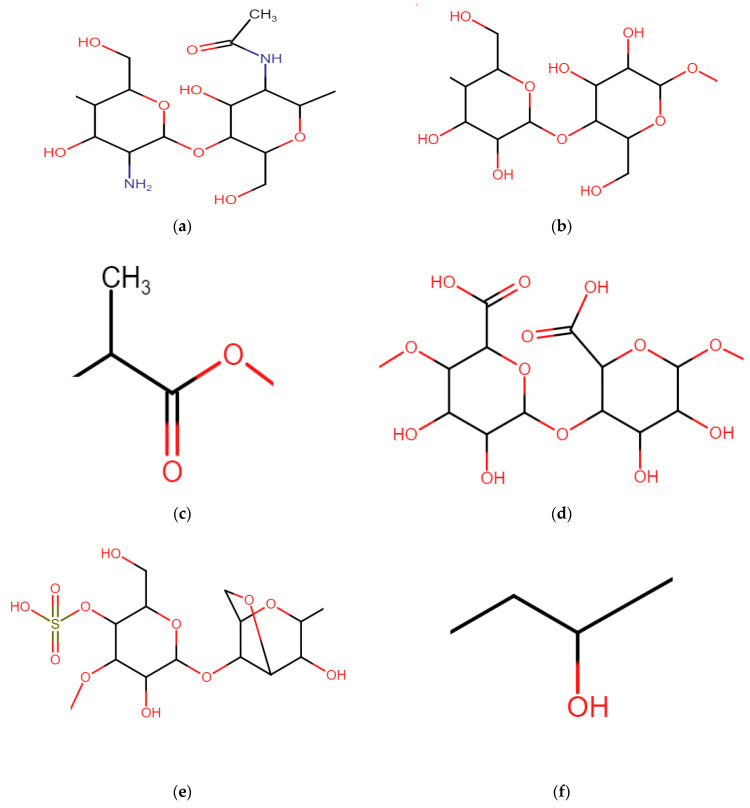
Molecular structure of (**a**) chitosan, (**b**) cellulose, (**c**) polylactic acid, (**d**) alginate, (**e**) k-carrageenan, and (**f**) polyvinyl alcohol.

Chitosan is a derivative of chitin and is the second most abundant biopolymer in the world. The chemical structure is shown in Figure 5. This polymer is hydrophilic, biodegradable and biocompatible. Furthermore, it has the potential to serve as a material for CEMs in fuel cells due to its thermal properties, good water retention and low reactants/methanol permeability. The disadvantages of chitosan are its low mechanical strength and high brittleness due to a high glass transition temperature. These limitations can be overcome by blending chitosan with other polymers or by chemical crosslinking. In addition, chitosan has low proton conductivity; sulfonation or phosphorylation is possible by reacting sulfonation or phosphorylation agents with chitosan’s -NH_2_ and -OH groups, which introduces functionality to the polymer. Typically, sulfonation is performed, using sulfonic acid, chlorosulfonic acid or sulfuric acid [18,19]. Binsu et al. [99] improved the properties of chitosan by blending it with polyvinyl alcohol (PVA) and adding phosphonic acid groups. Bijay et al. [100] did not introduce functional groups directly into the chitosan backbone, but they introduced aromatic rings containing carboxylic acid groups to chitosan, thereby increasing the proton conductivity. Furthermore, they created an organic–inorganic nanostructure by blending the modified chitosan with silica and PVA. They obtained high conductivities and low methanol permeabilities. Liu et al. [101] used silica-coated carbon nanotubes as the filler material to fabricate a chitosan-based composite CEM using sulfuric acid via a sol-gel method. The crosslinker reduced the water uptake of the membranes compared to that of chitosan membranes and significantly increased the oxidative stability, mechanical properties and proton conductivity (0.025 S/cm). However, they were not able to reach the conductivities of Nafion 117 (0.033 S/cm), and an excessively large increase in the number of silica-coated nanotubes led to proton conducting channels with higher tortuosities, eventually decreasing the conductivity. Finally, to evaluate the greenness and degree of sustainability of using chitosan, it is also important to study the production process of the biopolymer, which is in need of improvements. Chitosan is obtained by removing the acetyl groups from chitin. The major source of chitin is the shells of crustaceans, which are waste products of the seafood industry. The downside of this production process is that during the extraction process, multiple acid and alkaline treatments are performed that generate hazardous waste. In addition, the fluctuations in the availability of the feedstock makes it difficult to produce it on enormous scales. Efforts to make the extraction process greener are ongoing and make use of ionic liquids, deep eutectic solvents, microbial fermentation, enzyme-assisted extraction, microwave-assisted extraction, subcritical water extraction and electrochemical extraction [102,103].

Similar to chitosan, cellulose is very abundant on Earth. It is a basic element in plant fibers. Sources include rice husk, wheat husk, maize husk, pine needles and cotton. The extraction process of cellulose goes commonly via alkalization, bleaching, or acidic hydroloysis.These processes consist of three major steps: (i) pre-hydrolysis to open the matrix, for which mineral acid or alkali agents are used; (ii) pulping is the cooking of the fiber using NaOH; and (iii) bleaching is the final step in which H_2_O_2_, sodium chlorite and ozone are used [104]. Further research is necessary to remove some toxicity/hazards of the process in order to be able to speak of real sustainable cellulose. An interesting type of cellulose is bacterial cellulose. Bacterial cellulose is popular because it can be produced in a controlled environment by bacteria. However, at this point, the lack of understanding of the bacterial processes limits upscaling [105]. The chemical structure of cellulose is shown in Figure 5. Cellulose has interesting features for use in fuel cells, most of which are similar to chitosan. It has good thermal properties, good water retention, and low reaction permeability. Furthermore, it is biodegradable, has limited toxicity and is biocompatible. However, similar to chitosan, polymer blending and other modifications are necessary to synthesize CEMs with competitive performances [19]. Prior to sulfonation, an activation of the cellulose is necessary, and this can be carried out in different ways. Yue et al. [106] and Eldin et al. [107] performed the activation via immersion in NaIO_4_ and reaction with epichlorohydrin, respectively. Then, both investigations performed sulfonation by dipping it in an aqueous Na_2_SO_3_ solution. Besides the products needed to activate cellulose, the use of a Na_2_SO_3_ solution makes it a very interesting sulfonation process due to the low hazardous properties of sodium sulfite. In addition, sodium sulfite can also be produced in a relatively green way. It is formed by reacting SO_2_ with Na_2_CO_3_ using the lime dual alkali process. In spite of the acute toxicity of SO_2_, it does not have such important limitations because its purity does not need to be high. The purity of SO_2_ in flue gases from power plants, for example, is already high enough for Na_2_SO_3_ synthesis, making it possible to produce sodium sulfite while treating flue gases. Other products, such as NaHSO_3_ and Na_2_SO_4_, are also formed during this process, but the conditions can be controlled [108]. Bayer et al. [109] modified cellulose in a different way. They fabricated CEMs from cellulose nanofibers and cellulose nanocrystals. Cellulose nanocrystals are formed by dissolving the cellulose nanofibers in HCl or H_2_SO_4_ to dissolve the amorphous regions such that only the crystalline regions remain. In addition, the acid reacts with the hydroxyl groups to form sulfate esters, introducing functional groups to the polymer. They observed very low conductivities for the membranes synthesized from cellulose nanofibers, which is explained by the absence of functional groups. The membranes made from cellulose nanocrystals showed a higher conductivity (4.6 mS/cm at 120 °C and at 100% humidity). Both membranes had hydrogen gas permeabilities much lower than that of Nafion since they are crystalline and have a dense structure.

Polylactic acid (PLA) is obtained from agricultural products such as corn, sugar beet, starch and soy protein. Lactic acid monomer is produced from extracting sugar or starch from the aforementioned vegetable sources, mainly through fermentation. There are different steps to obtain PLA from starch, i.e., fermentation, electrodialysis, depolymerization, purification and polymerization. An interesting parameter to evaluate the greenness of the process of obtaining PLA from natural sources is via gross fossil energy [110,111]. The gross fossil energy is defined as the gross energy requirement minus the energy embodied in the renewable feedstock. The gross energy requirement is the total energy put in to obtain the biopolymer. The energy embodied in the feedstock represents the energy content of the renewable feedstock to produce a certain amount of useful biopolymer; it is defined by the heat of combustion of the feedstock and is fixed. For PLA, the gross energy and the gross fossil fuel energy to produce PLA from corn equals, respectively, 82.5 and 54.1 MJ/kg. This means that 54.1 MJ/kg of fossil energy is used to produce 1 kg of PLA. This value is 25–55% less than the fossil energy required for producing petroleum-derived plastics, making PLA a much greener option [110,111]. The structure of PLA is shown in Figure 5. PLA is part of the family of aliphatic polyesters and has many advantages such as low toxicity, low carbon footprint, high mechanical and thermal strength and biodegradability, which makes it useful for fabricating membranes as a modifier agent or main membrane matrix. To apply PLA for CEMs, there is a need to introduce additional functional groups since PLA only has carboxylic acid groups at the end of the polymer chain [97,112]. Cheng et al. [113] synthesized composite CEMs for the purification of lysozyme from chicken eggs via the incorporation of rice husk into a PLA matrix. Ion exchange capacities of 0.84 mmol/g were obtained (note that although this value approaches the value of Nafion 117, conductivity measurement should be performed to check the competitiveness). Xiong et al. [114] used a modified rice husk where the hydroxyl groups are partially deprotonated to enhance the presence of fixed anionic groups and thus the IEC and conductivity.

**Figure 6:**
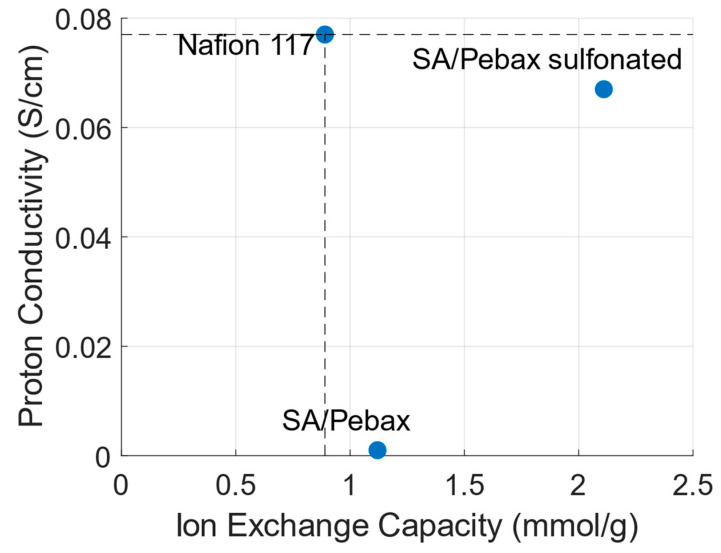
Proton conductivity vs. ion exchange capacity of the membranes synthesized and tested by Nagar et al. [116]. The low proton conductivity of the sodium alginate/Pebax (SA/Pebax) membranes that were not sulfonated show the need for sulfonation, despite the high ion exchange capacity.

Alginate is a natural linear polymer containing carboxylic acid groups. It is extracted from (brown) seaweeds via a multi-stage process consisting of mechanical treatment, soaking, acid and alkali pre-treatment and extraction. Furthermore, there are three precipitation routes to recover the alginate from the solution. The details of these process steps can be found in the review by Saji et al. [115]. More important is to ensure that the extraction process does not ruin the ‘greenness’ of working with alginate. Investigating the reported processes in the literature, around half of them use a 2% (*v*/*v*) formaldehyde solution to soak the seaweed. Formaldehyde is toxic and may cause cancer, even in small concentrations. Additionally, during the acid treatment, acidic solutions ranging from 0.1 to 5.5 M are used to remove non-targeted compounds and polyvalent cations [115]. Thus, improvements in these extraction processes can still be made to make it greener. The chemical structure of alginate is shown in Figure 5. Alginate consists of β-d-mannuronic acid and α-L-guluronic acid units and the ratio of these two units determines the gel strength. Alginate is an interesting biopolymer to synthesize CEMs due to its high biocompatibility, biodegradability, nontoxicity and low cost. Furthermore, since alginate is soluble in water, no organic solvents are necessary in the CEM synthesis process. A drawback is the low mechanical strength of alginate; therefore, it is often covalently or ionically crosslinked. Sodium alginate is the most common modification [98]. Nagar et al. [116] blended sodium alginate with a synthetic polymer Pebax, performed crosslinking with glutaraldehyde and sulfonated with sulfuric acid. They also investigated membranes that were not sulfonated with sulfuric acid. Figure 6 shows the observed IECs in relation to the measured proton conductivities. The dashed lines indicate the performance values of Nafion 117. They observed that both variants have ion exchange capacities (IECs) higher than that of Nafion 117 (1.12 mmol/g and 2.11 mmol/g versus 0.89 mmol/g). However, the IEC of the non-sulfonated membrane barely translates into any conductivity; it equaled 0.001 S/cm, whereas Nafion 117 has a conductivity of 0.077 S/cm. This shows the importance of sulfonating the membrane polymer despite the presence of carboxylic acid groups in the alginate backbone. The membrane that was sulfonated had a conductivity of 0.067 S/cm, which is close to the performance of Nafion 117. Smitha et al. [117] blended sodium alginate with deacetylated chitosan to use in direct methanol fuel cells. They observed that the deacetylated chitosan membrane already had a higher IEC than Nafion 117; however, its conductivity was approximately half (0.042 S/cm vs. 0.086 S/cm).

The final biopolymer with great potential for use in CEMs is carrageenan. Around 90% of the world’s carrageenan is produced by the red algae types, Kappaphycus and Eucheuma. The most important way to extract carrageenan from these red algae is via the combination of alkaline and heat extraction, followed by filtration and dehydration, thus using alkaline solutions such as NaOH, Ca(OH)_2_ or KOH [118]. Although this process makes use of hazardous alkaline solutions, the hazards of this extraction process remain limited. In addition, it is now possible to extract carrageenan using relatively eco-friendly technologies that focus on the use of water as an extraction agent [119]. Carrageenans are mainly used in the food industry as a gelling thickener and as a stabilizing agent. They are also used in cosmetics, paints, pharmaceuticals and the biomedical industry. k-Carrageenan is the most dominant carrageenan and contains a sulfate group (see Figure 5). It is an interesting polymer to make CEMs because of its high IEC, good film-forming ability and self-extinguishing properties. Disadvantages are poor mechanical properties and excessive solubility in water, which can be overcome by crosslinking [120]. Pahnavar et al. [120] prepared double network hydrogels based on a blend of modified k-carrageenan and polyvinyl alcohol and an acrylic acid matrix. They obtained good elongation to break of 71.8% and an IEC of 0.47 mmol/g, leading to an ionic conductivity of 0.0199 S/cm^2^. However, the water uptake was too high (70.7%). Also, Gouda et al. [121] chose to blend carrageenan with PVA and zirconium phosphate. Liew et al. [122] modified k-carrageenan with phosphoric acid to obtain higher conductivities, thank to thehigher number of oxygen atoms in the structure. These oxygen atoms serve as electron donor groups to form coordinated bonds with protons for transport. Eldin et al. [123] selected iota carrageenan instead of k-carrageenan because of its higher amount of sulfonic acid groups. Crosslinking was performed using glutaraldehyde and sulfophthalic acid. The latter crosslinking agent also contained sulfonic acid groups. In this way, a maximum ion exchange capacity of 1.2 mmol/g was obtained. Water was used as a solvent. The downside was the high water uptake; in addition, the proton conductivity and the oxidative stability were not determined.

Finally, some attention should be paid to (PVA) (see Figure 5), which is not a biopolymer but has multiple green aspects. It is an inexpensive polymer for which water can be used as a solvent, it is readily functionalized via the alcohol groups and is even partially biodegradable [124,125,126]. PVA-based CEMs can be synthesized using PVA as the main polymer matrix. The sulfonation of PVA is discussed above. Furthermore, PVA can easily be crosslinked with, for example, glutaraldehyde and succinic acid. These cross-linkers provide good control of the crosslinking degree and increase the hydrophobicity of the membrane matrix to improve the phase separation since PVA itself is hydrophilic [127,128,129]. In addition, this review provides multiple examples where PVA was used to prepare a polymer blend for CEMs.

In addition to the potential biopolymers that are discussed above to synthesize CEMs, many CEM polymers are in need of crosslinkers to improve properties such as mechanical strength and methanol permeability. Unfortunately, the use of green or sustainable crosslinkers is not common yet and other fields are using them in limited amounts. Tannic acid has the potential to be used as a green crosslinker in food packaging. It has good antibacterial and antioxidant properties and it improves the performance of a variety of biopolymer-based food packaging containing casein, gelatin, chitosan or PVA. The reactivity of tannic acid comes from its phenolic groups [130,131]. Furthermore, tannic acid is present in the coating layer on top of AEMs and CEMs [132,133]. Genipin is a second green crosslinker. In stem cell technology, genipin is used to crosslink alginate and chitosan to synthesize microcarriers [134]. Gorgieva et al. [135] fabricated nanocomposite membranes based on chitosan and genipin as a crosslinker. Du et al. [136] were able to replace glutaraldehyde with genipin to crosslink chitosan for the fabrication of pervaporation membranes for the dehydration of isopropanol. The membranes crosslinked using the green crosslinker obtained better permselectivities.

## 6. Green Solvents

**Figure 7:**
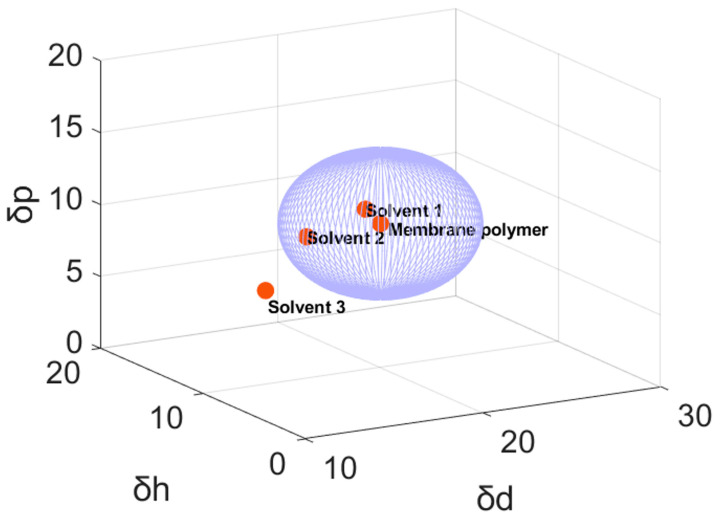
Representation of a Hansen space with a hypothetical membrane polymer and 3 hypothetical solvents. The sphere around the membrane polymer represents the space in which solvents should be located to have an affinity high enough to dissolve the polymer. Solvent 1 and 2 seem to be good solvents, but the solubility parameters of solvent 3 seems to deviate too much.

The solvents that are now commonly used are dimethylformamide (DMF), dimethylacetamide (DMAc) and n-methylpyrrolidon (NMP). Those solvents have the issue of being non sustainable, not green and very hazardous and toxic. They are included in the registration, evaluation, authorization and restriction of chemicals by the European Chemicals Agency who has the intention to ban them [137]. It is estimated that more than 5 billion liters of wastewater containing toxic solvents is produced annually due to membrane fabrication [138,139]. In addition, the solvent containing wastewater is in many cases incinerated because recovering the solvent is not straightforward [140,141]. These are incentives for switching to green and more sustainable solvents. There are multiple solvent properties that influence the performance of the membrane such as viscosity, dielectric constant, polarity and boiling point. (i) The higher the viscosity, the more difficult it is for the polymer to dissolve, which makes higher dissolution temperatures and times necessary. (ii) The degree to which a solvent can dissolve a polymer can be derived from Hildebrand’s solubility parameters. Every component has three solubility parameters, each corresponding to a certain energy. δ_h_ represents the energy from hydrogen bonds between molecules; δ_p_ corresponds to the contribution of dipolar intermolecular forces; and δ_d_ represents the energy of dispersion forces [142,143,144]. Polymers and solvents can be placed in the three-dimensional Hansen space, using the parameters as coordinates. The smaller the distance between the point of the polymer and the solvent, the better the solvent will dissolve the polymer. Figure 7 shows the Hansen space for a hypothetical membrane polymer and three hypothetical solvents. The solubility parameters of solvent 3 differ significantly from those of the membrane polymer and are, therefore, located far from the membrane polymer. (iii) The dielectric constant is a measure of the capacity of a chemical to store electrical energy. Therefore, solvents with a high dielectric constant are able to disperse ionic groups. For CEMs, this indicates a dispersion of the functional groups (sulfonic acid groups) [144,145]. (iv) If a solvent has a high boiling point, higher temperatures are needed if the CEMs are fabricated via solvent evaporation. However, low-volatility solvents have the safety advantages of having a higher flash point and an easier control of possible releases to the environment.

Ma et al. [144] investigated the influence of the solvent solubility parameters and dielectric constant on the microstructure of the membrane, more specifically on the formation of possible aggregates for PVDF membranes. They were able to explain the membrane performance parameters to the type of aggregates formed. They observed that the solubility parameter had the greatest influence. The more similar the solubility parameter of the solvent is to the hydrophobic backbone of Nafion, the better the polymer dissolution, leading to less aggregation. Therefore, the polymer formed larger aggregates when methanol/water mixtures were used instead of DMAc or DMF. The higher the dielectric constant, the better the dissociation of the sulfonic acid groups, leading to smaller aggregates due to charge repulsion. Therefore, the aggregates formed when using N-methyl formamide are smaller when methanol/water is used as the solvent, but larger than those formed with DMAc or DMF. DMF’s solubility parameter is not similar to the solubility parameter of the Nafion backbone, but it has a high dielectric constant. Finally, they correlated the larger aggregates to higher conductivity, and also to higher water and methanol permeability. Fontanavova et al. [145] prepared CEMs from sPEEK-WC (sulfonated polyetheretherketone) using DMAc and DMSO while changing the degree of sulfonation, the nature and concentration of inorganic additives, casting temperature and membrane post treatment. They came to the same conclusion, i.e., using DMSO leads to membranes with higher methanol permeabilities and higher conductivities. Kaliaguine et al. [146] found a disadvantage of using a solvent that is very similar to that of the polymer. They used DMAc and DMF to fabricate CEMs from sulfonated PEEK. DMF formed strong hydrogen bridges with sulfonated PEEK, leaving behind traces on the membrane polymer, resulting in a conductivity lower than when DMAc was used [146]. However, this result should be looked at with great care since more recent studies have proven that DMF can be the optimal solvent to make sulfonated PEEK membranes [147]. Xi et al. [147] found that DMF was a better solvent than DMF, DMAc, NMP and DMSO to make CEMs for vanadium redox flow batteries. The different conclusions compared to the work of Kaliaguine et al. might be explained by the residual sulfuric acid. Kaliaguine et al. explain that the residual sulfuric acid on the membrane can react with DMF and DMAc at high temperatures. Sulfuric acid is difficult to remove from highly sulfonated PEEK and can, therefore, influence the results. In addition, Kaliaguine et al. state that the conductivity measurement technique can also be a reason for the difference in conductivity results. Blending solvents is a way to alter the solubility parameters. Lu et al. [148] dissolved polysulfon (PSF) in a blend of Rhodiasolv^®^ PolarClean and γ-Valerolactone (GVL). The δ_p_ of Rhodiasolv^®^ PolarClean is the closest to the δ_p_ of PSF and the δ_d_ and δ_h_ of GVL are the closest to the δ_d_ and δ_h_ of PSF; therefore, blending the two solvents reduces the distance in the Hansen space and creates a better dissolution.

Without a doubt, the best solvent to synthesize CEMs in terms of safety and sustainability is water. As previously discussed, certain biopolymers and PVA can be used as main membrane polymer material. However, if other polymers need to be dissolved, there is the need for organic solvents.DMSO is the most successful low-hazardous solvent for making CEMs at this moment. DMSO has a very low acute toxicity; thus, it is generally considered a safe chemical [149]. In addition, the industrial production uses lignin, which is a byproduct of the pulping process, to produce DMSO [142]. Unfortunately, to the best of our knowledge, no other low-hazardous green solvent besides DMSO have been used to fabricate CEMs. The environmental impact and health hazards show the seriousness of this finding. In addition, the number of studies using DMSO to fabricate CEMs is limited. DMSO has been mainly used to fabricate CEMs based on sulfonated PEEK [145,150]. Neither attempt was able to compete with the performance of the commercial Nafion membranes. Another study by Li et al. [52] used DMSO as a solvent to sulfonate poly(4,4′-diphenyl ether-5,5′-bibenzimidazole) with sulfonated sodium styrene. They also used DMSO to dissolve the sulfonated polymer in order to obtain a 12 wt% casting solution. To allow the development of CEM synthesis procedures using green solvents, inspiration can be taken from other membrane fields. In those fields, Cyrene™, Rhodiasolv^®^ PolarClean, TamiSolve NxG and γ-valerolactone have shown potential. Rhodiasolv^®^ PolarClean is derived from a by-product of Nylon 6,6 fabrication and commercialized by Solvay Novecar [148]. It is classified as a green, low-hazardous solvent; however, it knows multiple obstacles in its synthesis process that need to be overcome [151]. γ-valerolactone is produced by the processing of hemicellulose and cellulose [148]. Cyrene™ (dihydrolevoglucosenone) is produced from the pyrolysis of cellulose-containing biomass. It is a safe chemical and it is biodegradable, without the emission of SO_x_ and NO_x_ [152]. TamiSolve NxG (*N*-butylpyrrolidinone) is considered one of the next-generation green solvents. It is a non-reprotoxic and biodegradable solvent and it can be used as an alternative to NMP for multiple applications [142]. Although these solvents are already applied in the membrane field, no study has been found where they are used to fabricate CEMs. To implement it in the fabrication of CEMs, the lessons learned in the other membrane fields can serve as a starting point. Therefore, Table 2 contains an extensive overview of how the solvents are used in other fields.

## 7. Conclusions and Future Prospects

Human society has never influenced the world as much as it does today. Negative consequences are present, such as air pollution, climate change, alterations in water quality and aquatic biodiversity and incidents involving toxic chemicals. In order for future generations not to be harmed, it is necessary to live in a more thoughtful and creative harmony with nature. Technologies such as water electrolysis, electrodialysis and artificial photosynthesis will play a major role in the transition to a more sustainable society. Since these technologies make use of CEMs, it is crucial that the synthesis of this material can be carried out in a green/sustainable way. Perfluorinated membranes are the most commonly used today because of their high conductivity and high stability. Their synthesis, however, is far from sustainable, leading to large volumes of acidic waste streams, water pollution, incidents with fatal casualties and a contribution to climate change. This review discussed in detail the role of perfluorinated polymers in the present PFAS crisis. Furthermore, a summary of the patents published on the synthesis of these polymers was provided to identify the sustainability and safety problems. At present, the search for greener/sustainable cation exchange membranes has been unsuccessful. This review gathers for the first time all the steps involved in the synthesis of CEMs and critically evaluates the alternatives that could enable the transition towards a more sustainable processing. A division was made based on the different aspects of CEM synthesis, i.e., sulfonation, selection of polymers and solvent. The findings of this review can be used as a guideline in the development of future CEMs.

Regarding sulfonation, the traditional oxidative agents, such as fuming sulfuric acid, sulfuric acid and chlorosulfonic acid, do not meet the green principles of green chemistry due to their hazardous characteristics and acid waste generation. The use of SO_3_ as a sulfonating agent is a greener choice because no acid waste is generated. However, operating sulfonation processes with liquid SO_3_ causes practical problems such as a quick increase in viscosity and considerable heat generation. To avoid this problem, SO_3_ can be dissolved in a solvent, and then a green solvent should be selected to comply with the principles of green chemistry. Another possibility would be to perform the sulfonation with gaseous SO_3_. A second alternative to the traditional oxidative agents is the use of already sulfonated monomers. However, the manner in which these monomers are made determines the ‘greenness’ of the process. In the majority of cases, the use of traditional oxidative agents is still based on the origin of these monomers. Another way to introduce functional groups is via the use of filler materials. Filler materials are already used in CEMs to increase chemical–and thermal stability. Sulfonating the filler materials leads to enhanced conductivities due to the more efficient conducting networks resulting from their lower tortuosity. This can make the sulfonation procedure greener by performing interfacial plasma due to the use of a lower H2SO4 concentration. Furthermore, carbon nanotubes show the highest potential in the journey towards greener membrane fabrication.

The review discussed in detail green alternatives for the conventional polymers to make the membrane polymer. Chitosan, cellulose, polylactic acid, alginate and carrageenan show the highest potential. In addition, polyvinyl alcohol has also been discussed in detail due to its low toxicity and the possibility of using water as a solvent. These polymers are all biocompatible with low toxicity. In addition, many of them have good thermal stability and methanol rejection. However, these polymers need to be adjusted by means of sulfonation or blending with other polymers to enhance their performance to competitive levels, which, eventually determines the ‘greenness’ of the membrane. Furthermore, tannic acid and genipin can be considered green crosslinkers for the membrane polymer.In the last part of the review, the transition towards green solvents is presented. Several solvents already in use in the membrane field, among which DMSO, Cyrene™, Rhodiasolv^®^ PolarClean, TamiSolve NxG and γ-valerolactone, could be considered applicable. They are not considered toxic, and most of them are produced from sustainable resources. However, to date, none of these solvents is specifically employed in the fabrication of CEMs. This review shows findings present in other membrane fields that could be used as a starting point for a more responsible fabrication of CEMs.

## Figures and Tables

**Table 1:** List of the 12 principles of green chemistry of Anastas and Warner and the 10 objectives of the United Nations for green and sustainable chemistry [20,27].

12 Principles of Green Chemistry of Anastas and Warner
Waste prevention: Better avoid the waste than treat it afterwards.
Atom economy: Use the maximum number of reagent atoms in your product to reduce waste.
Less hazardous chemical synthesis: Minimize substances’ hazards during reactions and waste.
Designing safer chemicals: Minimize the toxicity directly by molecular design.
Safer solvents and auxiliaries: Choose as few as possible solvents and auxiliaries and choose the safest solvent.
Design for energy efficiency: Choose the least energy-intensive chemical route.
Use of renewable feedstocks: Use as much as possible renewable sources and as few as possible petrochemical sources.
Reduce derivatives: Minimize the use of temporary derivatives such as protecting groups.
Catalysis: Use catalytic reactions to help increase selectivity, minimize waste and reduce reaction times and energy demands.
Design for degradation: Design chemicals that can degrade and thus be discarded easily.
Real-time pollution prevention: Monitor the processes in real time to prevent the release of hazardous and polluting substances.
10 objectives of green and sustainable chemistry of the United Nations
Minimizing Chemical Hazards: Design of chemicals with minimized (or no) hazard properties for use in materials, products and production processes (“benign by design”).
Avoiding regrettable substitutions and alternatives: Develop safe and sustainable alternatives for chemicals of concern through material and product innovations that do not create negative trade-offs.
Sustainable sourcing of resources and feedstocks: Use of sustainably sourced resources, materials and feedstocks without creating negative trade-offs.
Advancing Sustainability of Production Processes: Use green and sustainable chemistry innovation to improve resource efficiency, pollution prevention, and waste minimization in industrial processes.
Advancing Sustainability of Products: Use green and sustainable chemistry innovation to create sustainable products and consumption with minimized (or no) chemical hazard potential.
Minimize chemical release and pollution: Reduce chemical releases throughout the life cycle of chemicals and products.
Enabling non-toxic circularity and minimizing waste: Use of chemistry innovations to enable non-toxic circular material flows and sustainable supply and value chains throughout the life cycle.
Maximizing Social Benefits: Consider social factors, high standards of ethics, education and justice in chemistry innovation.
Protecting workers, consumers, and vulnerable populations: Safeguard the health of workers, consumers and vulnerable groups in formal and informal sectors.
Developing solutions for sustainability challenges: Focus on chemistry innovation to help address societal and sustainability challenges.

**Table 2:** Overview of green solvents used in other membrane fields.

Solvent	Main Polymer	Brief Description	Refs.
Cyrene™	PES and PVDF	Porous membranes via phase inversion	[153]
	PSF	Dense asymmetric gas separation membranes	[154]
	Cellulose, CA ^1^	Membrane via phase inversion	[155]
Cyrene and Cygnet 0.0 ^2^	CA, PSF and polyimides	Flat sheet membranes using pure cyrene, pure cygnet or a blend of the two.	[156]
DMSO	PES	Ultrafiltration and nanofiltration membranes	[157,158]
	OPBI ^3^	Sulfonation step performed with DMSO and CEM cast from a 12 wt% casting solution	[52]
	PEEK-WC	Comparison of DMAc and DMSO and varying multiple membrane synthesis parameters	[145]
	Nafion polymer	Influence of solubility parameters and dielectric constant	[144]
	sPEEK	Synthesis CEM	[146,150]
GVL	CA, CTA ^4^, PI, PES and PSF	Membrane preparation via phase inversion	[159]
TamiSolve NxG	PES	Synthesis of porous membranes	[138,160]
	PVDF-HFP ^5^	Porous membranes for membrane distillation	[143,161]
	PVDF	Porous membranes	[162,163]
	PEEK	Membrane for organic solvent nanofiltration	[164]
	PSF	Synthesis of a crosslinked support	[165]
	PEEK-WC	Synthesis catalytic membranes	[166]
Rhodiasolv^®^ PolarClean	PES	Ultrafiltration and nanofiltration membranes	[167]
	PES, PSF and CA	Ultrafiltration and nanofiltration membranes	[168]
	Matrimid^®^	Microfiltration and ultrafiltration membranes	[169]
Polarclean and GVL	PSF	Ultrafiltration membranes	[170]
	PES and PET	Ultrafiltration membranes	[14]

^1^ Cellulose acetate; ^2^ cygnet 0.0 is a derivative of cyrene and is produced via the reaction of cyrene and ethylene glycol; ^3^ poly(4,4’-diphenyl ether-5,5’-bibenzimidazole); ^4^ cellulose triacetate; ^5^ polyvinylidene fluoride-hexafluoropropylene.

## Data Availability

Data are contained within the article.
